# Supplementary material for: Implicit pattern learning predicts individual differences in belief in God in the United States and Afghanistan
Source: Nat Commun. 2020 Sep 9;11:4503. doi: 10.1038/s41467-020-18362-3 (PMC7481241; doi:10.1038/s41467-020-18362-3)
Supplement: Supplementary file 1 — Supplementary Information [file 41467_2020_18362_MOESM1_ESM.pdf]

Supplementary Information for:

Implicit Pattern Learning Predicts Individual Differences in Belief in God in the United States and Afghanistan

Weinberger et al., 2020

Supplementary Methods

1. Data quality control

Substantial data loss/exclusion for the Afghan sample was anticipated because issues related to cross-cultural relevance and comprehension are common for data collection in Afghanistan[1]. Local experimenters were hired to conduct data collection sessions with local participants in Kabul, consistent with best practices based on Z.W.'s extensive experience collecting data in Afghanistan, e.g., as director of the Asia Foundation's Survey of the Afghan People[1]. Also consistent with best practices, we implemented additional post-collection quality control protocols to ensure that all data were properly collected and recorded by the local experimenters. This involved screening of the data to identify any irregularities associated with individual experimenters, reviewing experimenter notes for problems with study participants, and interviews with experimenters after the study to determine whether participants properly understood tasks, and whether the experimenters themselves had properly understood and implemented all study procedures.

We received data for 354 participants in Afghanistan. The majority of data collection occurred on site at the Afghan Cultural House in Kabul. Upon reviewing the data, we identified irregularities in data collection for two local experimenters (survey and task responses collected by these experimenters showed very low variability within and across participants, and response times were consistently different from the rest of the sample). Because these two experimenters were not able to satisfactorily account for their data, we excluded all 157 participants from whom these two experimenters collected data (all during the final months of the collection period). Two additional participants were excluded because notes at the time of the study indicated that they were responding dishonestly and/or that they were uncooperative with study procedures (e.g., one

participant expressed unwillingness to cooperate because they perceived that they were "working with Westerners").

Among the 195 remaining participants, 8 were excluded from analyses for failing to finish the survey (i.e., they did not reach the end of the survey) and 9 were removed for improper completion times on the survey measures (defined as < 25 minutes or > 4 hours). An additional 14 participants were excluded because of inappropriate responding during the SRTT (defined as average RTs on the SRTT of >2000ms or <100 ms). In accordance with principles of informed consent and the Georgetown University institutional review board, participants were permitted to skip questions as desired. Sixteen participants did not complete key measures related to the hypotheses addressed by the current study (i.e., SRTT, IB measures, covariate personality/belief variables), and were thus excluded from analyses. After these exclusions, the final Afghan sample was 148 participants.

In the U.S. sample, the initial cohort of 240 was reduced to 199 for the following reasons. Five participants were removed because they did not comply with protocol for the testing session. Five participants did not complete the SRTT and two participants did not complete all of the belief measures. An additional 29 participants experienced a malfunction in the stimulus presentation during the SRTT. Specifically, the first random block consisted of only 22 targets (rather than 50). Theoretically, the reduced number of trials and task duration might have diminished participants' opportunity to increase their speed of responding over the course of the block. These 29 participants were conservatively removed from the primary analyses reported in the main text.

The online sample (i.e., European sample, Supplementary Table 2) was recruited through *Prolific*. Recent evidence indicates that *Prolific* offers higher quality data (e.g., based on participant attention and honest responding) and a more diverse participant pool compared to alternative online research platforms, such as Amazon Mechanical Turk[2,3]. The survey contained

two attention check questions embedded within the study measures (e.g., “Blue is virtually always the same color as orange?”). Twelve participants did not correctly answer both of these attention check questions and were excluded from analysis. One additional participant was excluded for being under 18 years of age at the time of the study. The constituency of this sample was primarily (83%) European (Supplementary Table 2).

In sum, the vast majority of all exclusions were related to either experimenter unreliability (in the Afghan sample) or a malfunction in stimulus presentation (in the U.S. sample). The impact of all of these decisions on the results reported in the main text are displayed in Supplementary Tables 10, 11. Results were largely unchanged across different inclusion/exclusion criteria, indicating that these inclusion/exclusion determinations did not substantially impact the observed findings.

2. Additional study measures

We sought to develop an interventionist belief outcome variable that could be investigated in both the U.S. and Afghanistan. The tasks that were ultimately included in the IB variable were drawn from established measures with clear scoring procedures, and were deemed by Z.W. and local Afghan experimenters to have been successfully translated and culturally adapted (i.e. they were properly understood by experimenters and participants in Afghanistan). Several other religious belief measures were developed or adapted in-lab to be assessed for criteria of scoreability, as well as for translatability and cultural adaptability in Afghanistan, but were ultimately excluded on at least one (and generally all) of these criteria. These measures were:

- ***A “drawing task,***” in which participants were asked to free-draw a rendering of God’s involvement in an event.
- ***A “story task,***” in which participants answered questions about the implied role of a deity in an audio narrative.

- ***A “heatmap/proximity task,***” in which participants indicated by mouse click the proximity of God’s power to a depicted event (a scoring method was developed based on pixel-wise proximity to the corner of the screen closest to the depicted event).
- **“Single item change in belief measure”** in which participants indicated whether their strength of belief in God increased, decreased, or stayed the same since childhood.

The story task and drawing task were excluded from data collection in Afghanistan due to substantial issues with both scoreability and cultural adaptability. Administration of the lab-developed audio narrative stimuli in the story task was also determined by experimenters to be somewhat problematic in the U.S. sample, e.g., optimal responding placed substantial demand on sustained auditory attention (which appeared to vary between participants), and favored native English speakers, suggesting potential confounds. The “heatmap/proximity” task was included in Afghan data collection but ultimately deemed by experimenters to have been poorly understood. Including the scores for the story task and the heatmap/proximity task (either separately or together) in the IB component score did not substantially change any of the results for any sample in which these measures were collected (e.g., IL-pat remained predictive of IB, all Ps < 0.02). We elected to exclude these measures in the U.S. sample primarily so that the IB scores would be constituted by the same measures in the two samples. The single item change in belief measure was omitted because scores were redundant with those obtained from the richer Belief Change measure discussed in Section 4 and in the main text. This was confirmed in both the U.S. and Afghan samples via Spearman’s rank correlations between the single item measure and the full Belief Change measure, as well as ANOVA models in which participants’ Belief Change scores were grouped by their single item responses (all $P < .0001$). Thus, the single item measure, which was included primarily as a simpler alternative in case the full Belief Change measure was unsuccessful, served as a confirmatory check that the full Belief Change measure was properly understood.

Participants also completed other measures/tasks related to separate cross-cultural hypotheses concerning causality perception as well as personality and cognitive characteristics in U.S. and Middle Eastern cultural contexts. These were the following:

- Locus of Control[4]
- Coincidence Frequency[5]
- Unusual Experiences subscale of Short Boundaries Questionnaire[6]
- Magical Ideation[7]
- Teleological Bias[8]
- Santa Clara Strength of Religious Faith Questionnaire[9]
- "Belief – Doubt," a lab-developed measure for which participants responded to 2 questions intended to assess their confidence and doubt that God exists. One of these questions was analyzed as an "Existence Belief" variable in exploratory analyses (see section 5).
- Reaction Time Task[10]
- Michotte Launching Task[11]
- Pulling Squares; Separating Motion (lab-developed tasks for causality perception, based on prior work[12,13])
- Mental Rotation Task[14]

The results reported in the main text describe a schizotypal thinking measure, which was used as a personality-trait covariate because it has been shown to be related to both religious belief[15] and biases to perceive order[16,17]. Magical Ideation, Unusual Experiences, Coincidence Frequency, and Locus of Control (Externality dimension) were all highly collinear with schizotypal thinking, and were deemed likely to estimate related personality constructs. Thus, they were excluded from use in the present study. Experimenters also expressed concerns about the language and cultural translation/understanding of many of these tasks for the Afghan sample, including the teleological bias scale, for which an ultimately unsuccessful attempt was made at a completely re-written version in Dari, the lab-developed existence belief item, which is discussed in detail in Section 5, and the SCSRFQ, which is largely focused on Christian-specific elements of belief that are not relevant in Islam. An attempt to modify the Reaction Time Task, which focuses on belief in fictional/supernatural characters (e.g., Bilbo Baggins) and elements of primarily Christian belief (e.g., salvation), was not deemed to have sufficiently broadened the scope for use across multiple

religious samples (including non-Christian religious affiliations within the U.S.), and necessarily compromised the validity of the English-language scale. Note that the Reaction Time Task, the Existence Belief item, and the SCSRFQ, which estimates involvement in and personal importance of religion, are also conceptually distinct from measures of belief in an intervening god that were used to calculate IB.

Exploratory analyses indicated that the choice of which covariate to include in the regression models had no effect on the associations between IL-pat and religious belief. The effect of IL-pat on IB with schizotypy and parents' belief as covariates (U.S.: $\beta = 0.17$, $P = 0.009$; Afghanistan: $\beta = 0.26$, $P = 0.001$), as reported in the manuscript, was not meaningfully different from the effect of IL-pat within alternative models using any individual covariate in place of schizotypy (U.S.: βs $= 0.15 – 0.20$, Ps = .002 – .019; Afghanistan: βs $= 0.21 – 0.25$, Ps = 0.003 – 0.007) or with any additional covariate used in conjunction with schizotypy and parents' belief (U.S.: βs $= 0.15 – 0.20$, Ps = .002 – .018; Afghanistan: βs $= 0.23 – 0.25$, Ps = 0.002 – 0.003). Similarly, IL-pat was as predictive in the primary Belief Change model (U.S. $\beta = 0.15$, $P = 0.036$; Afghanistan: $\beta = 0.17$, $P = 0.036$) as in all alternative models in which schizotypy was replaced with a different covariate (U.S.: βs = 0.15 – 0.16, Ps = 0.03 – 0.04; Afghanistan: βs = 0.16 – 0.17, Ps = 0.04 – 0.056). When each covariate was used in conjunction with schizotypy and parents' belief, IL-pat remained a significant predictor of Belief Change in all models (U.S.: βs = 0.15 – 0.16, Ps = 0.03 – 0.04; Afghanistan: βs = 0.17, Ps = 0.04 – 0.046), with one exception (magical ideation in the U.S.: $\beta = 0.13$, $P = 0.059$, which was highly collinear with schizotypy: $r = 0.64$).

3. IB component score

Participant responses on the BDIS (Supplementary Table 3) and Overlapping Circles tasks (Supplementary Fig. 1) were used to create an Interventionist Belief (IB) principal component score (Supplementary Fig. 2, Supplementary Table 8) for each participant using a least squares regression approach such that scores for each sample had a mean of 0 and a standard deviation of 1. In all 3 samples (U.S., Afghan, European), all interventionist belief variables were strongly correlated with each other (all Ps < 0.001; Supplementary Tables 5-7). To confirm that the results presented in the main text were not simply a by-product of this data reduction method (i.e., PCA, orthogonal components), we standardized (z-score) scores on all 3 measures in all samples and then averaged them together to obtain another estimate of interventionist belief. The two approaches yielded scores that were highly correlated (all $r > 0.99$, $P < 0.0001$).

Supplementary Results

4. Belief Change

Belief Change was surveyed using a lab-developed change of belief measure, which consisted of 9-point Likert scales on which participants reported their own strength of belief in God starting at age 6 and then at three-year intervals up to age 24. Instructions stated that participants should not respond to items beyond their present age. Asking participants to report belief at three-year intervals was intended to encourage more precise and thorough consideration of responses than can be achieved by a single item measure (e.g., “Has your belief changed since childhood?”). This method to assess change in religious belief is consistent with prior work that has demonstrated the efficacy of assessing religiosity retrospectively[18–21].

To quantify change in belief, the primary outcome of interest was a *difference score* in which strength of belief in God at age 6 (the youngest age reported) was subtracted from the

strength of belief at the oldest reported age. Exploratory analysis was also conducted for a *slope of change score* in which a line of best fit (using the polyfit function in MATLAB) was calculated for each participant, using each age for which they reported a strength of belief as the X variables and the corresponding belief as the Y variables. Thus, these two metrics distinguish between *how much* belief changed since age 6 (i.e., difference score) and *the rate at which* this change occurred (i.e., slope of change score). We favored the difference score approach because 1) amount of change provided a more clearly interpretable outcome measure than slope of change – for example, it was not clear what differences in components of slope would mean given equivalent amounts of change, or how differences in slope might be considered in relation to other variables of interest (e.g., IL-pat); 2) We were wary of issues related to fine-grained accuracy of retrospective data (e.g., accurately distinguishing between belief at age 6 vs. age 9); and 3) We were also mindful of potential "anchoring"[22] at the ends of the scale based on strong recollections of belief in early childhood and current age (e.g., reporting levels of belief at intermediate ages to indicate regular progression between the youngest and current age).

Exploratory analyses indicated that difference and slope scores were strongly associated with one another in both samples ($r > 0.95$, $P < 0.0001$), thus the two Belief Change outcomes were likely to reflect subtle differences in estimation rather than conceptually distinct psychological phenomena. Further, a series of post-hoc tests (chi-square) following simultaneous linear regression models in which both Belief Change scores were regressed on IL-pat, schizotypal thinking, and parents' strength of belief in both the U.S. Afghan samples (using the seemingly unrelated estimate command in STATA) yielded no significant differences in the effect sizes of IL-pat on Belief Change (U.S.: $\chi^2 = 0.26$, $P = 0.61$; Afghanistan: $\chi^2 = 0.06$, $P = 0.80$), and IL-pat was associated with Belief Change in all four models (all Ps $\leq .05$). Thus, the strength of the

coefficients for IL-pat as a predictor of Belief Change did not differ meaningfully for the difference score vs. the slope score. Taken together, these results suggest that, while the effect size of IL-pat on change in belief from childhood to adulthood is relatively modest, this effect is robust to differences in the Belief Change score used.

5. Existence Belief

Participants responded to two questions intended to measure the difference between belief vs. doubt in the existence of a God. Responses were made on a 9-point Likert scale:

- Question 1: "Rate the strength of your current belief in God" (1= do not believe God is real at all, 9 = absolutely certain God is real)
- Question 2: "Rate the strength of your doubt, if any, that God is real" (1= no doubt at all, 9 = maximum doubt).

We initially planned to score this "Belief-Doubt" measure by subtracting Item 2 from Item 1, to obtain a potentially more nuanced assessment of belief in the existence of God than what is obtainable from a single item. However, responses to these two items mirrored each other to such a strong extent ($r = -.87$, $P < 0.0001$) that this measure was essentially reducible to a single item. Further, because subtracting doubt from belief could yield identical scores for individuals with different strength of belief that God exists (e.g., 7 for Question 1 and 5 for Question 2 vs. 3 for Question 1 and 1 for Question 2), we elected to focus only on Question 1 to estimate participants' "Existence Belief" for the analyses described below.

We anticipated a strong, positive correlation between Existence Belief and IB, which was a much richer variable and much more directly related to our primary hypothesis concerning implicit pattern learning and belief in the ordering influence of God, and we anticipated that any

association between IL-pat and Existence Belief would be a largely redundant reflection of the association between IL-pat and IB (see below). Additionally, the Existence Belief measure was determined to be non-viable in the Afghan sample. No acceptable translation for Muslim Dari speakers was found to convey the intended sense of belief (i.e., specifically relative to doubt) with respect to the existence of God, and experimenters reported that participants found questions concerning uncertainty that God exists to be insulting and/or "dangerous." By contrast, questions concerning the level of interventionist belief were possible because they did not involve an implication of non-existence. Thus, even if it had been theoretically desirable, it would not have been possible to include Existence Belief in the models involving equivalent variables in the two main samples. Nonetheless, supplementary analyses were conducted to investigate the relationship of Existence Belief to other key variables in the U.S. sample only.

As expected, Existence Belief was positively correlated with each of the three individual measures of interventionist belief (all *r*s > 0.47, *P*s < 0.0001) and with the Interventionist Belief component score (IB; $r = 0.66$, $P < 0.0001$). In order to quantify differences between Existence Belief and IB, we rescaled both variables such that scores for both variables would range from -1 to 1. Because belief that God exists is presumably a prerequisite for belief in God as an intervening influence, average IB would not be expected to exceed Existence Belief (i.e., those reporting belief in an intervening God would generally be expected to believe that God exists, but the reverse might not be true). Paired t-tests using the rescaled belief measures supported this prediction; participants held significantly lower IB ($M = -0.19$, $SE = 0.04$) than Existence Belief ($M = 0.06$, $SE = 0.05$; $t(198) = -7.07$, $P < 0.0001$). That is, participants reported higher belief that God exists relative to their belief in God as an intervening influence. Further, we identified a marked difference in the distribution of responses for Existence Belief and IB (Supplementary Fig. 3; one-sample

Kolmogorov–Smirnov: $P < 0.001$). In particular, we observed a large clustering of participants who reported little to no belief in an intervening God (i.e., a positive skew for IB). However, the majority of participants *did* endorse an Existence Belief, and this variable was characterized by a fairly bi-modal distribution. These differences suggest that a subset of participants believed in a god, but one that is relatively non-interventionist.

Exploratory analyses to examine whether Existence Belief was associated with IL-pat revealed no correlation ($r = 0.05$, $P = 0.50$). Thus, while IB and Existence Belief are strongly correlated, only the former was associated with IL-pat. However, because Existence Belief was closely related to IB in some participants (but not in others), we next asked whether IL-pat was more associated with Existence Belief in individuals for whom IB more closely approximated Existence Belief and less associated with Existence Belief in those for whom Existence Belief was more divergent from IB (putatively explaining the discrepant relationships of IL-pat to IB vs. Existence Belief). In order to do this, we first calculated a "Belief Difference" score by subtracting rescaled Existence Belief from rescaled IB (a score of 0 indicated equivalent Existence Belief and IB; positive values indicated stronger IB relative to Existence Belief). To explore whether IL-pat differentially predicted Existence Belief based on how closely Existence Belief related to IB, we interacted the absolute value of Belief Difference with IL-pat to predict Existence Belief. We used the absolute value of Belief Difference because our question concerned similarity (i.e., how close to 0 is the difference between the two belief variables) rather than relative magnitude. This model revealed a significant Belief Difference X IL-pat interaction, such that the association between IL-pat and Existence Belief was stronger for those participants whose IB more closely approximated Existence Belief ($\beta = -0.20$, $P = 0.002$). In other words, the strength of the relationship between IL-pat and Existence Belief was related to the similarity between IB and Existence Belief. Taken

together, these post-hoc analyses demonstrate that, consistent with our hypothesis concerning IL-pat as a contributor to IB, IL-pat was more predictive of Existence Belief in participants for whom the level of existence belief was closely tied to the level of IB, and less predictive in those whose belief in God appeared to be less tied to belief in the intervening influence of God (i.e., those who report higher levels of Existence Belief relative to IB). One plausible interpretation is that IL is more strongly related to Existence Belief in a subset of individuals for whom belief that God exists is largely focused and/or dependent on belief in the interventionist influence of God, and less related to Existence Belief in others for whom belief in the existence of God depends more on other factors.

The above analyses indicated that the extent to which IL-pat was predictive of Existence Belief varied by how closely Existence Belief was related to IB (i.e., Belief Difference). A second regression model in which the absolute value of Belief Difference was interacted with IL-pat and regressed on Belief Change revealed results similar to those described above. Individuals with more similar Existence Belief and IB showed a stronger association between IL-pat and Belief Change (US: $\beta = -0.16$, $P = 0.024$), indicating that the effect of IL-pat on Belief Change may also be largely dependent on the extent to which belief in God reflects belief in the interventionist influence of God.

In contrast to Existence Belief, however, IL-pat *was* predictive of Belief Change in the full sample. Additional analyses were performed to elucidate the reason for this distinction. First, although Existence Belief and Belief Change were positively correlated ($r = 0.54$, $P < 0.0001$), a paired t-test indicated a significant difference between rescaled Existence Belief (scores from -1 to 1; $M = 0.06$, $SE = 0.05$) and rescaled Belief Change ($M = -0.05$, $SE = 0.03$; $t(196) = -2.47$, $P = 0.014$).

Additionally, the association between Belief Change and Existence Belief was not uniform across the full range of Existence Belief (Supplementary Fig. 4), reflecting the fact that the Belief Change measure was sensitive to the trajectory of belief (i.e., increase, decrease, amount of change) whereas Existence Belief was not. Participants who decreased in strength of belief since childhood were overrepresented at the low end (i.e., 1-3) of the Existence Belief scale (because it was not possible to increase in belief to reach the lowest levels of Existence Belief). Similarly, participants who increased in strength of belief since childhood were overrepresented at the high end (i.e., 7-9) of the Existence Belief scale (because it was not possible to decrease in belief to reach the highest levels of Existence Belief). However, the middle Existence Belief group (i.e., 4-6) contained a wide range of change in belief values (both positive and negative change).

To explore this further, we divided the sample into 3 groups based on Existence Belief (Low = 1-3, Moderate = 4-6, High = 7-9). A one-way ANOVA revealed significant differences in Belief Change (z-scored) between these groups ($F(196) = 46.59$, $P < 0.0001$), with only the High belief group showing above-average Belief Change. We also observed differences in the relationship between Existence Belief and Belief Change. Most notably, in the Low belief group (N = 69), Existence Belief and Belief Change were anticorrelated ($r = -0.26$, $P = 0.03$), and in the Moderate belief group (N = 47) there was no association between Existence Belief and Belief Change ($r = -0.08$, $P = 0.60$). Indeed, in the low belief group, participants with the highest Existence Belief (i.e., 3; average Belief Change = -.96, SE = .14) actually *decreased* in belief significantly *more* since childhood relative to those with the lowest Existence Belief (i.e., 1; average Belief Change = -0.39, SE = 0.21; $t(46) = 2.25$, $P = 0.03$), and in the Moderate belief group, participants with the lowest Existence Belief (i.e., 4; average Belief Change = 0.25, SE = 0.37) increased in belief nominally

more than participants with the highest Existence Belief (6; average Belief Change = -0.09, SE = 0.17).

The data thus indicate against a superficial prediction that Belief Change should straightforwardly track with Existence Belief (i.e., that those who increase more in belief should have higher Existence belief whereas those who decrease more should have lower Existence Belief). In fact, it is evident that this was frequently not the case (e.g., individuals who decreased in belief frequently had higher Existence Belief than other individuals who increased in belief). Inferences based on these statistical tests and nominal comparisons should be treated with some caution due to small and/or imbalanced sample sizes, but the data suggest that Belief Change is distinct from – and at times inversely related to – Existence Belief. The overall pattern of results (non-linear relationships, negative values for Belief Change verses positive values for Existence Belief) indicate that sensitivity to the trajectory of belief, which was an attribute of the Belief Change variable but not the Existence Belief variable, is an empirically meaningful basis for differentiating these variables in relation to each other and in relation to IL-pat.

6. Linear regression model fit

As described in the main text, two linear regression models in each sample were run to test our primary hypotheses that IL-pat predicted (1) IB, and (2) Belief Change. Each model controlled for schizotypal thinking and parents' strength of religious belief in order to separate effects of IL-pat from key psychological and environmental factors that are associated with religious belief[15,23] and biases to perceive order[16,17] but distinct from the hypothesized influence of IL-pat. Linear regression models accounted for significant variance in IB (U.S.: $F(3,195) = 17.01$, $P < 0.0001$, adjusted $R^2 = 0.20$; Afghanistan: $F(3,144) = 11.72$, $P < 0.0001$, adjusted $R^2 = 0.18$) and Belief

Change in Afghanistan ($F(3,138) = 5.71$, $P = 0.001$, adjusted $R^2 = 0.09$), and a small amount of variance in for Belief Change in the U.S. ($F(3,193) = 2.01$, $P = 0.11$, adjusted $R^2 = 0.02$).

7. Ordered logits to predict religious belief DVs

Because of non-normal distributions of IB – and the large cluster around 0 for Belief Change (Supplementary Fig. 2) – we ran ordered logistic regressions in both samples to confirm that the findings reported in the main text were not due to a violation of OLS assumptions.

Interventionist Belief Groups. For IB, we used a quartile split to create 4 groups corresponding to *low belief* (U.S.: $M_{IB} = -1.04 \pm 0.11$; Afghan: $M_{IB} = -1.36 \pm 0.20$); *medium-low belief* (U.S.: $M_{IB} = -0.59 \pm 0.15$; Afghan: $M_{IB} = -0.44 \pm 0.34$), *medium-high belief*, (U.S.: $M_{IB} = 0.21 \pm 0.30$; Afghan: $M_{IB} = 0.60 \pm 0.22$), and *high belief* (U.S.: $M_{IB} = 1.47 \pm 0.49$; Afghan: $M_{IB} = 1.19 \pm 0.20$).

Belief Change Groups. For Belief Change, we created 3 group corresponding to *decrease* (U.S.: $M_{Change} = -4.25 \pm 1.94$; Afghan: $M_{Change} = -3.09 \pm 1.67$), *small decrease/no change* (U.S.: $M_{Change} = -0.29 \pm 0.46$; Afghan: $M_{Change} = 0.00 \pm 0.00$), and *increase* (U.S.: $M_{Change} = 2.66 \pm 1.61$; Afghan: $M_{Change} = 3.56 \pm 2.66$).

Results. The results of these models (Supplementary Table 9) were largely comparable with those obtained using linear regression. Because of issues associated with creating a categorical variable from a continuous measure, we decided to focus on the results of the linear regression in the main text.

8. Mediation analyses

Results reported in the main text are consistent with an *a priori* model in which intuitions of universal order mediate the effect of IL-pat on IB and Belief Change (in the U.S. sample). While the mediation model was developed on theoretical grounds rather than empirical comparisons of alternative models, we performed additional exploratory analyses to in which alternative models were compared. While absolute model fit statistics are not applicable to these just-specified models, it is possible to derive the proportion of the total effect that is mediated in each model.

First, we tested fully reversed mediation models in which the belief measures were positioned as the independent variables and IL-pat was positioned as the dependent variable (i.e., Belief → UO → IL-pat; Supplementary Fig. 6.) The model in which IB was the independent variable yielded a nonsignificant indirect effect (β = 0.07, P = 0.08; bootstrapped bias-corrected 95% CI, as compared to β = 0.07, P = 0.005 in the original model) and a smaller proportion of the total effect that was mediated (0.35 compared to 0.48 in the original model). The model in which Belief Change was the independent variable yielded an indirect effect that remained marginally significant but also accounted for a smaller proportion of the total effect (P = 0.047; proportion of total effect that was mediated = 0.38 compared to 0.43 in the original model). A model in which IB was positioned as the mediator (rather than the dependent variable) and UO was positioned as the dependent variable (instead of the mediator; Supplementary Fig. 7) yielded an indirect effect (β = .087, P = .022, as compared to β = .090, P = 0.005 in the original model), and the proportion of the total effect that was mediated was again smaller than in the original model (0.44 compared to 0.48 in the original model).

Further exploratory analyses tested whether UO mediated the effects of IL-pat on each of the three individual belief measures from which the IB component score was derived (BDIS, Self Overlap, and World Overlap). These analyses revealed significant indirect effects in each case (all

Ps ≤ 0.006, bootstrapped bias-corrected 95% CI). By contrast, models in which the mediator and DV were reversed for each of these belief measures (such that each belief measure was positioned as the mediator, with UO as the dependent variable), revealed nonsignificant indirect effects for the BDIS and World Overlap variables, and smaller proportions of the total effect mediated for all three models (BDIS = 0.33; World Overlap = 0.30; Self Overlap = 0.36) compared to what was observed with UO as the mediator (BDIS = 0.73; World Overlap = 0.43; Self Overlap = 0.37). Similarly, the fully reversed mediation models with each individual IB measure positioned as the independent variable and IL-pat as the dependent variable yielded a nonsignificant indirect effect for the Self Overlap model, and smaller proportions of the total effect mediated for all three models (BDIS = 0.65 p. = .017; World Overlap = 0.36 p. = .032; Self Overlap = 0.28) than in the above-described models with IL-pat as the independent variable and each of the three IB measures as dependent variables. Thus, taken as a group, these exploratory analyses suggest modest but consistent empirical support for the hypothesized models relative to alternatives models.

a

The diagram below is designed to represent your image of how much God directly affects your actions. Please click and drag the left circle ("my actions") in an appropriate relationship with the right circle ("God") so that the overlap represents your image of how much God directly causes your actions. More overlap represents that God plays a more direct role in causing your actions. No overlap indicates that God has no direct role in causing your actions (this answer is appropriate if you do not believe God is real).

b

The diagram below is designed to represent your image of how much God affects events in the world. Please click and drag the left circle ("events in the world") in an appropriate relationship with the right circle ("God") so that the overlap represents your image of how much God affects events in the world. More overlap represents that God plays a more direct role in causing events. No overlap indicates that God has no direct role in causing events (this answer is appropriate if you do not believe God is real).

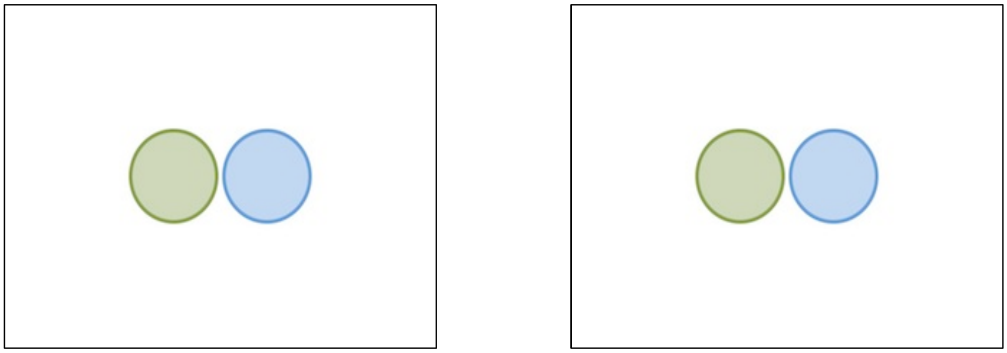

Supplementary Figure 1. Overlapping circles tasks. Participants move circles to reflect influence of God. Participants completed one task pertaining to God and their own actions (A) and one task pertaining to God and events in the world (B). Greater overlap is interpreted as stronger belief in an intervening god.

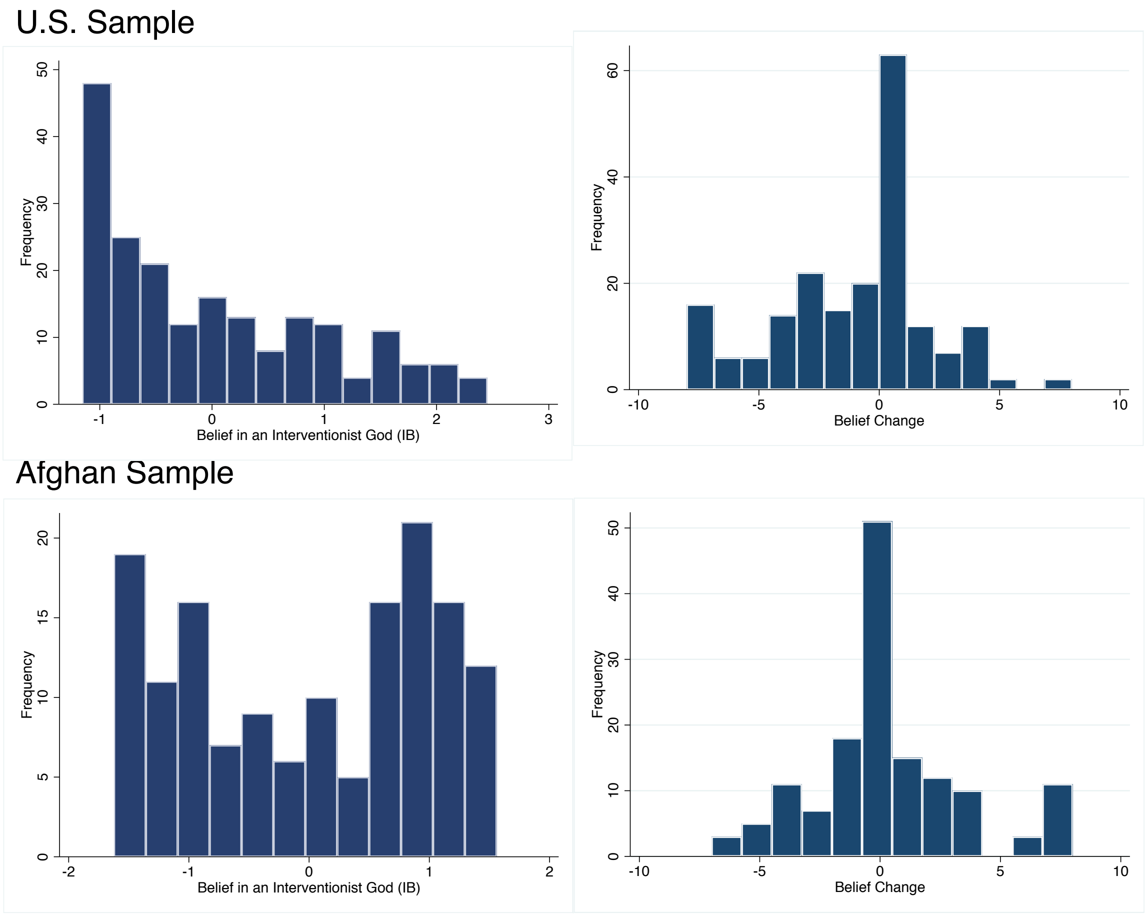

Supplementary Figure 2. Distribution of belief variables. Belief in an interventionist god (left) was calculated from a principal component analysis of the two Overlapping Circles tasks and BDIS.

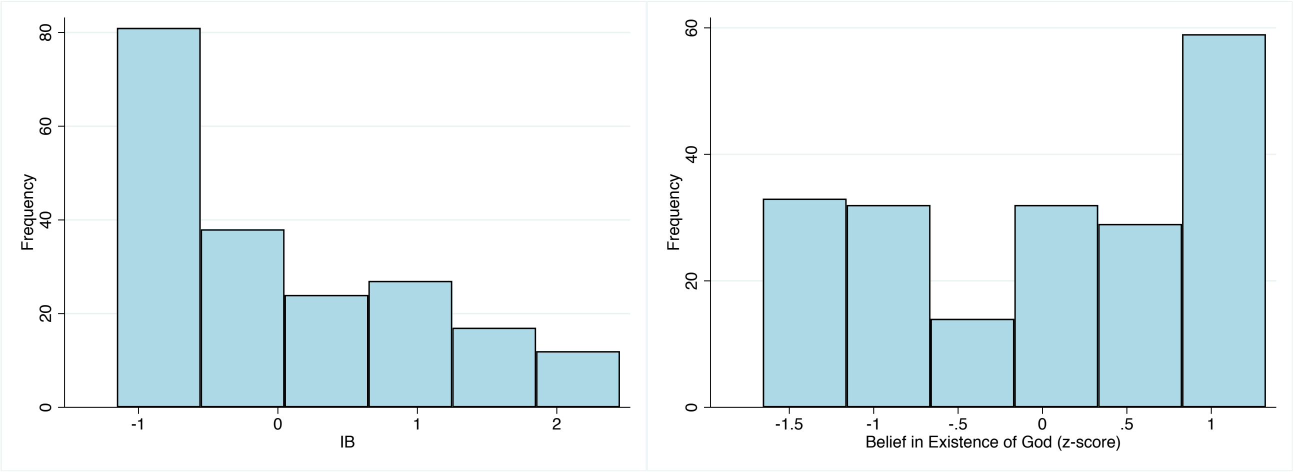

Supplementary Figure 3. Distribution of Existence Belief and IB. IB was characterized by a positive skew whereas Existence Belief was more bimodal (Kolmogorov-Smirnov indicated significant differences in distribution at $P < 0.001$, uncorrected). Paired t-tests revealed higher Existence Belief than IB (two-sided $P < 0.0001$, uncorrected).

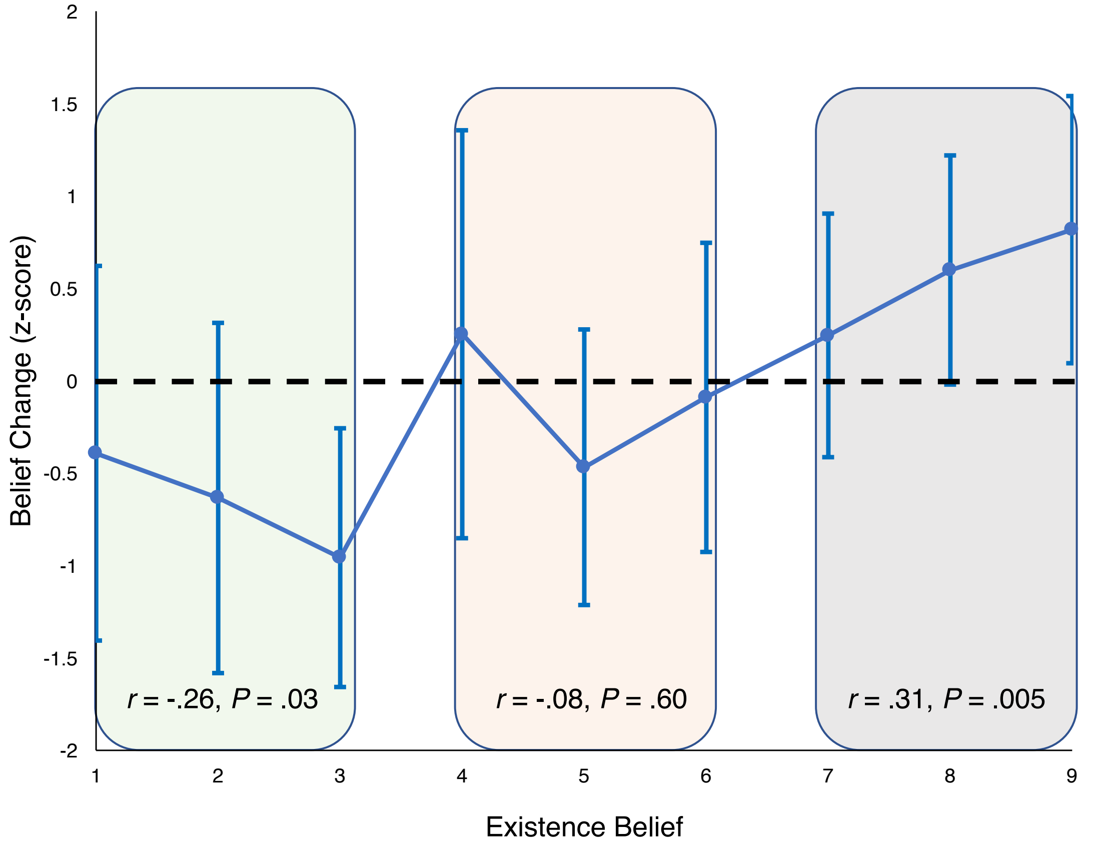

Supplementary Figure 4. Belief Change varies in its association with Existence Belief. For those in the low belief group (left box), Belief Change was negatively associated with Existence Belief. There is no association at moderate belief (middle box) and a positive relationship for those with high belief (right box). Points indicate mean Belief Change value (z-scored) for each Existence Belief group; SD indicated.

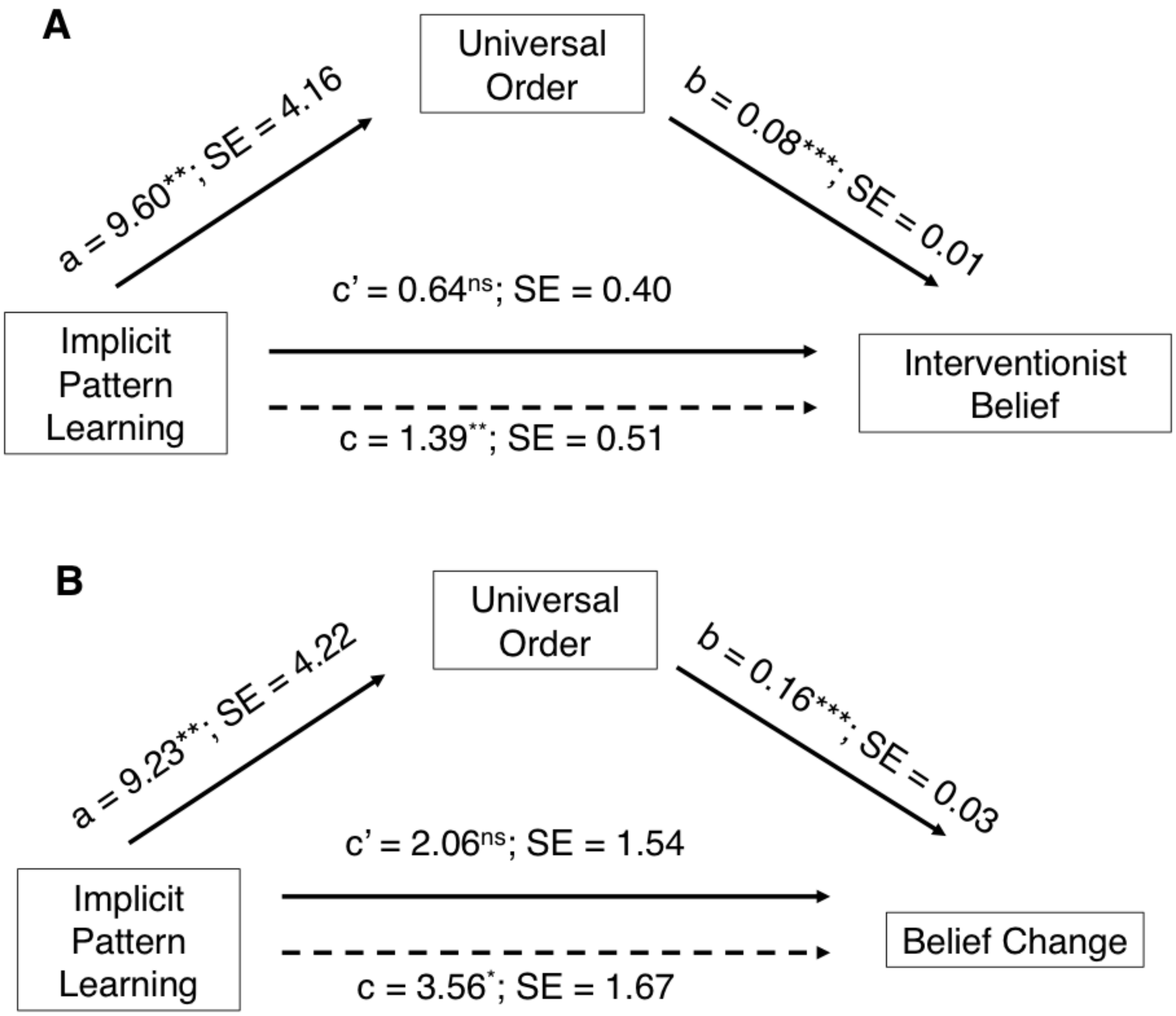

Supplementary Figure 5. Intuitions of universal order (UO) mediates effects of implicit pattern learning (IL-pat) on religious belief outcomes – 4-item UO estimation. Models for a) IB and b) Belief Change used UO estimates from all four items. Results are consistent with those reported in the main analyses.

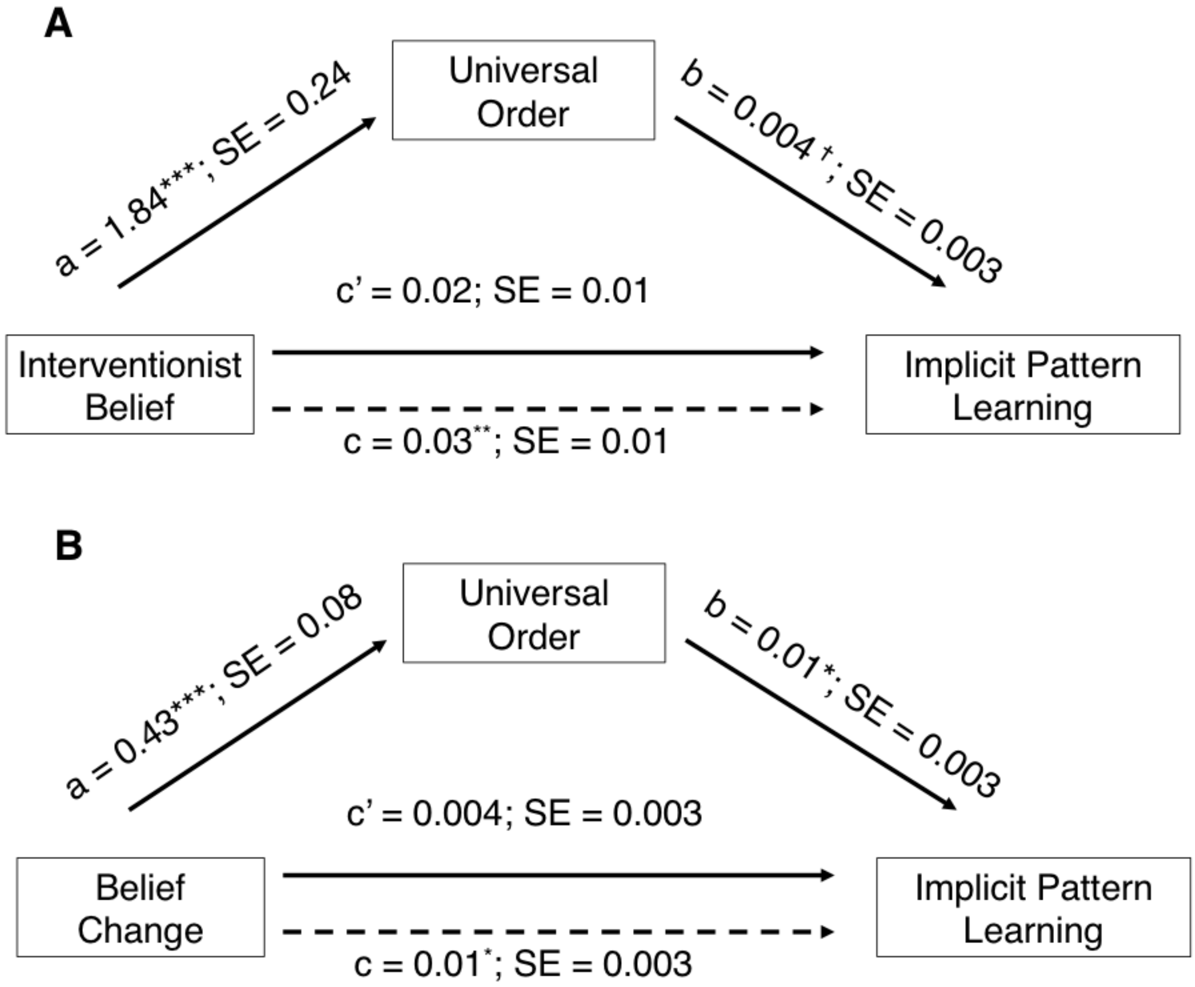

Supplementary Figure 6. Fully reversed mediation model. Alternative models position religious belief variables of a) IB, and b) Belief Change as the IV and implicit pattern learning as the DV. Mediated effects are nonsignificant (a) and weaker (b) than those indicated in Fig. 3 in the main text.

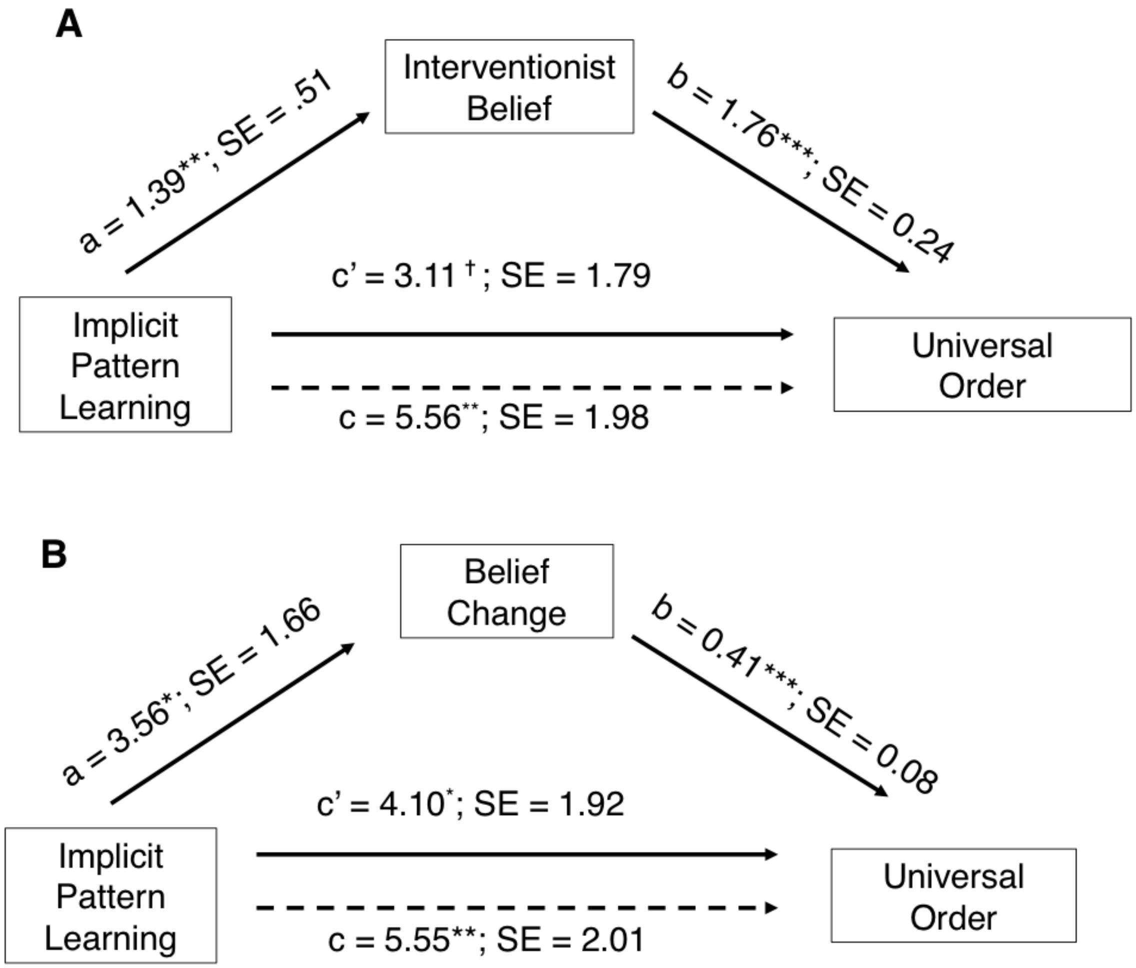

Supplementary Figure 7. Alternative mediation models. Alternative models positioned religious belief variables of a) IB, and b) Belief Change as the MV and UO as DV. Evidence of mediation is nominally weaker than in the theoretically-based model (Fig. 3) described in the main text.

Supplementary Table 1. Religious affiliations of U.S. participants during childhood and at the time of study.

Religion Raised	n	Percent	Religion Now	n	Percent
Christian	140	70.35%	Christian	104	52.26%
Roman Catholic (79)			*Roman Catholic (54)*		
All others (61)			*All Others (50)*		
None	27	13.57%	None	50	25.13%
Hindu	8	4.02%	Agnostic/Atheist	17	8.54%
Jewish	8	4.02%	Jewish	7	3.52%
Mixed	8	4.02%	Mixed	5	2.51%
Other	8	4.02%	Other	16	8.04%
Total	*199*		*Total*	*199*	

Note: All religious affiliation information was obtained by free-response.

Supplementary Table 2. Geographic constituency of online sample ("European sample")

	N	Percent
**Europe**	**80**	**83.33%**
Belgium (1)		
Denmark (1)		
Finland (1)		
France (1)		
Georgia (1)		
Germany (1)		
Greece (2)		
Hungary (2)		
Italy (4)		
Latvia (1)		
Poland (19)		
Portugal (19)		
Sweden (1)		
Switzerland (1)		
United Kingdom (25)		
**North America**	**12**	**12.50%**
United States (8)		
Mexico (4)		
**Asia**	**1**	**1.04%**
Israel (1)		
**Africa**	**3**	**3.12%**
South Africa (3)		

Supplementary Table 3. Belief in Divine Intervention Scale.

"Please indicate your agreement or disagreement with the following statements, from Strongly Disagree to Strongly Agree"						
	-3 (Strongly disagree)	-2	-1	1	2	3 (Strongly agree)
1. God sometimes directly intervenes to heal individuals of diseases like cancer.						
2. God sometimes communicates directly with individuals.						
3. God does not intervene directly in our lives						
4. God sometimes directly intervenes to change the course of damaging weather conditions like hurricanes.						
5. Real miracles of healing from God do not occur today.						
6. God sometimes uses dreams to communicate with us.						

Note: Items 3 and 5 are reverse scored.

Supplementary Table 4. Universal Order survey questions

"Please indicate your level of agreement or disagreement with the following statements:"									
	1<br>Disagree	2	3	4	5<br>Neutral	6	7	8	9<br>Agree
1. Everything happens for a reason									
2. There is order to the universe									
3. There is a plan that guides events in the universe									
4. Something beyond physics plays a part in deciding what happens									

Note: Items 3 and 4 were excluded from calculation of UO variable because of an implied reference to a god. The European sample was presented with only Items 1 and 2.

Supplementary Table 5. Zero-order correlations between IL-pat and religious belief variables in U.S. sample

	IL-pat	IB	BDIS	Self Overlap	World Overlap
IB	0.19<br>(0.008)	--			
BDIS	0.13<br>(0.06)	0.81<br>(<0.001)	--		
Self Overlap	0.20<br>(0.006)	0.91<br>(<0.001)	0.59<br>(<0.001)	--	
World Overlap	0.16<br>(0.02)	0.90<br>(<0.001)	0.57<br>(<0.001)	0.78<br>(<0.001)	--
Belief Change	0.15<br>(0.03)	0.43<br>(<0.001)	0.46<br>(<0.001)	0.38<br>(<0.001)	0.32<br>(<0.001)

Note: P values indicated parenthetically for IL-Pat correlations; two-sided, uncorrected

Supplementary Table 6. Zero-order correlations between IL-pat and religious belief variables in Afghan sample

	IL-pat	IB	BDIS	Self Overlap	World Overlap
IB	0.23<br>(0.004)	--			
BDIS	0.19<br>(0.02)	0.79<br>(<0.001)	--		
Self Overlap	0.19<br>(0.02)	0.95<br>(<0.001)	0.60<br>(<0.001)	--	
World Overlap	0.26<br>(0.001)	0.95<br>(<0.001)	0.60<br>(<0.001)	0.93<br>(<0.001)	--
Belief Change	0.16<br>(0.06)	0.47<br>(<0.001)	0.53<br>(<0.001)	0.37<br>(<0.001)	0.38<br>(<0.001)

Note: P values indicated parenthetically for IL-Pat correlations; two-sided, uncorrected

Supplementary Table 7. Zero-order correlations between belief variables in European sample.

	IB	BDIS	Self Overlap
BDIS	0.87	--	
Self Overlap	0.86	0.59	--
World Overlap	0.90	0.69	0.67

Note: all $P < 0.0001$, two-sided, uncorrected

Supplementary Table 8. Loadings on first component (PCA) in all samples

	Loading	Uniqueness
**U.S. Sample**		
BDIS	0.81	0.34
Self Overlap	0.91	0.17
World Overlap	0.90	0.18
**Afghan Sample**		
BDIS	0.79	0.37
Self Overlap	0.95	0.10
World Overlap	0.95	0.10
**European Sample**		
BDIS	0.87	0.25
Self Overlap	0.86	0.26
World Overlap	0.90	0.19

Supplementary Table 9. Ordered logit regression models to predict Interventionist Belief and Belief Change

	U.S. Sample						Afghan Sample					
	Odds Ratio	SE	z	P	95% Conf. Interval		Odds Ratio	SE	z	P	95% Conf. Interval	
**Interventionist Belief**												
IL-Pat	10.92	11.00	2.37	0.018	1.52	78.60	22.49	21.83	3.21	0.001	3.36	150.70
Schizotypy	2.57	1.65	1.47	0.142	0.73	9.03	18.11	12.03	4.36	0.000	4.92	66.58
Parents' Belief	1.32	0.07	5.10	0.000	1.19	1.47	1.25	0.21	1.30	0.194	0.89	1.74
**Belief Change**												
IL-Pat	6.20	6.24	1.81	0.070	0.86	44.54	8.31	8.77	2.01	0.045	1.05	65.82
Schizotypy	0.75	0.49	-0.44	0.657	0.20	2.72	5.23	3.32	2.61	0.009	1.51	18.16
Parents' Belief	0.94	0.05	-1.11	0.266	0.85	1.04	1.15	0.20	0.80	0.422	0.82	1.60

Supplementary Table 10. Impact of QC data inclusion/exclusion on IB, Belief Change, and Universal Order in U.S. sample

	Interventionist Belief					Belief Change					Universal Order				
	β	B	SE	t	P	β	B	SE	t	P	β	B	SE	t	P
**Manuscript Sample (N=199)**															
IL-Pat	0.17	1.24	0.47	2.65	0.009	0.15	3.53	1.67	2.11	0.036	0.18	5.13	1.90	2.69	0.008
Schizotypy	0.11	0.53	0.30	1.75	0.082	-0.07	-1.10	1.08	-1.02	0.311	0.06	1.06	1.23	0.86	0.393
Parents' Belief	0.38	0.15	0.03	5.96	0.000	-0.04	-0.05	0.09	-0.53	0.595	0.28	0.42	0.10	4.10	0.000
**No QC (N=233)**															
IL-Pat	0.18	1.31	0.43	3.02	0.003	0.15	3.53	1.53	2.31	0.022	0.19	5.50	1.74	3.16	0.002
Schizotypy	0.14	0.64	0.28	2.32	0.021	-0.05	-0.78	0.97	-0.80	0.425	0.12	2.15	1.11	1.94	0.054
Parents' Belief	0.39	0.15	0.02	6.60	0.000	-0.04	-0.05	0.08	-0.68	0.499	0.28	0.41	0.09	4.51	0.000
**No SRTT QC (N=228)**															
IL-Pat	0.17	1.25	0.43	2.89	0.004	0.15	3.52	1.55	2.27	0.024	0.19	5.25	1.76	2.99	0.003
Schizotypy	0.13	0.62	0.28	2.22	0.027	-0.06	-0.89	0.99	-0.90	0.371	0.12	2.23	1.13	1.97	0.050
Parents' Belief	0.39	0.15	0.02	6.63	0.000	-0.05	-0.06	0.08	-0.73	0.468	0.28	0.41	0.09	4.51	0.000

Supplementary Table 11. Impact of QC data inclusion/exclusions on IB and Belief Change in Afghan sample

	Interventionist Belief					Belief Change				
	β	B	SE	t	P	β	B	SE	t	P
**Manuscript Sample (N=148)**										
IL-Pat	0.26	1.60	0.48	3.33	0.001	0.17	3.53	1.67	2.11	0.036
Schizotypy	0.35	1.40	0.30	4.72	0.000	0.26	3.37	1.04	3.24	0.001
Parents' Belief	0.09	0.11	0.09	1.22	0.225	0.10	0.38	0.31	1.25	0.212
**No QC (N=176)**										
IL-Pat	0.21	1.38	0.46	3.00	0.003	0.16	3.23	1.52	2.12	0.036
Schizotypy	0.33	1.29	0.28	4.69	0.000	0.20	2.50	0.92	2.73	0.007
Parents' Belief	0.05	0.06	0.08	0.69	0.489	0.10	0.35	0.28	1.27	0.207
**No SRTT QC (N=162)**										
IL-Pat	0.24	1.51	0.47	3.21	0.002	0.16	3.24	1.62	2.00	0.047
Schizotypy	0.35	1.37	0.28	4.86	0.000	0.23	2.91	0.97	3.00	0.003
Parents' Belief	0.10	0.11	0.09	1.29	0.200	0.10	0.39	0.30	1.29	0.200
**No Questionnaire QC (N=162)**										
IL-Pat	0.23	1.45	0.47	3.10	0.002	0.18	3.50	1.57	2.23	0.027
Schizotypy	0.33	1.31	0.29	4.52	0.000	0.23	2.88	0.97	2.95	0.004
Parents' Belief	0.04	0.05	0.08	0.60	0.547	0.10	0.35	0.28	1.24	0.217

Supplementary Table 12. Regression models to predict Interventionist Belief in both samples with additional explicit SRTT covariate regressors

	U.S. Sample					Afghan Sample				
	β	B	SE	t	P	β	B	SE	t	P
**Explicit Accuracy**										
IL-Pat	0.18	1.32	0.47	2.80	0.006	0.26	1.62	0.52	3.10	0.002
Schizotypy	0.12	0.55	0.30	1.81	0.073	0.36	1.46	0.34	4.35	0.000
Parents' Belief	0.38	0.15	0.03	5.93	0.000	0.05	0.06	0.09	0.61	0.545
Explicit Accuracy	0.09	0.26	0.18	1.41	0.160	0.04	0.12	0.23	0.52	0.603
**Illusory Pattern Detection**										
IL-Pat	0.17	1.24	0.47	2.63	0.009	0.27	1.68	0.52	3.25	0.001
Schizotypy	0.11	0.53	0.30	1.73	0.086	0.35	1.40	0.33	4.20	0.000
Parents' Belief	0.38	0.15	0.03	5.87	0.000	0.11	0.15	0.11	1.33	0.187
Illusory	0.04	0.07	0.12	0.58	0.561	0.10	0.17	0.14	1.16	0.247
**"Yes bias"**										
IL-Pat	0.17	1.27	0.48	2.68	0.008	0.27	1.71	0.52	3.28	0.001
Schizotypy	0.11	0.54	0.31	1.77	0.078	0.36	1.45	0.33	4.40	0.000
Parents' Belief	0.39	0.15	0.03	5.97	0.000	0.11	0.14	0.11	1.29	0.201
Yes bias	-0.03	-0.03	0.08	-0.45	0.653	0.05	0.05	0.08	0.65	0.518

Supplementary Table 13. Regression models to predict Belief Change in both samples with additional explicit SRTT covariate regressors

	U.S. Sample					Afghan Sample				
	β	B	SE	t	P	β	B	SE	t	P
**Explicit Accuracy**										
IL-Pat	0.16	3.69	1.68	2.20	0.029	0.18	3.73	1.83	2.03	0.044
Schizotypy	-0.07	-1.05	1.08	-0.98	0.331	0.32	4.25	1.18	3.61	0.000
Parents' Belief	-0.04	-0.05	0.09	-0.56	0.577	0.06	0.22	0.32	0.68	0.497
Explicit Accuracy	0.07	0.64	0.65	0.99	0.323	-0.01	-0.11	0.79	-0.14	0.891
**Illusory Pattern Detection**										
IL-Pat	0.15	3.46	1.67	2.08	0.039	0.18	3.73	1.84	2.03	0.044
Schizotypy	-0.08	-1.14	1.07	-1.07	0.288	0.31	4.20	1.19	3.53	0.001
Parents' Belief	-0.05	-0.06	0.09	-0.68	0.496	0.06	0.28	0.39	0.72	0.475
Illusory	0.12	0.71	0.43	1.64	0.104	0.00	0.00	0.51	0.00	0.999
**"Yes bias"**										
IL-Pat	0.14	3.32	1.68	1.97	0.050	0.18	3.74	1.84	2.03	0.045
Schizotypy	-0.08	-1.18	1.08	-1.09	0.276	0.31	4.20	1.17	3.58	0.001
Parents' Belief	-0.05	-0.06	0.09	-0.64	0.525	0.06	0.28	0.39	0.72	0.474
Yes bias	0.08	0.31	0.26	1.18	0.241	0.00	0.00	0.29	0.01	0.996

Supplementary Table 14. Zero-order correlations for U.S. re-contact sample

	CRT Correct	CRT Intuitive	Belief in Science	IL-Pat	IB	Universal Order
CRT Intuitive	-0.87*					
	(<0.001)					
Belief in Science	-0.03	0.03				
	(0.82)	(0.82)				
IL-Pat	-0.07	-0.03	-0.28			
	(0.56)	(0.84)	(0.02)			
IB	-0.10	0.140	-0.22	0.45		
	(0.41)	(0.27)	(0.07)	(<0.001)		
Universal Order	0.04	-0.01	-0.19	0.39	0.47	
	(0.76)	(0.95)	(0.13)	(0.001)	(<0.001)	
Belief Change	0.06	-0.13	-0.28	0.39	0.57	0.38
	(0.64)	(0.32)	(0.02)	(0.002)	(<0.001)	(0.002)

Note: Top row displays *r* values for correlations, *P* values (uncorrected) indicated parenthetically. All tests two-sided.

Supplementary Table 15. Zero-order correlations for online European sample

	IB	Universal Order	Belief in Science	CRT Analytic
Universal Order	0.37<br>(0.01)			
Belief in Science	-0.28<br>(0.06)	-0.22<br>(0.15)		
CRT Analytic	-0.19<br>(0.21)	-0.50<br>(0.001)	0.16<br>(0.29)	
CRT Intuition	0.21<br>(0.17)	0.46<br>(0.001)	-0.09<br>(0.57)	-0.87<br>(<0.001)

Note: Top row displays *r* values for correlations, *P* values (uncorrected) indicated parenthetically. All tests two-sided.

Supplementary Table 16. Linear regression results in U.S. re-contact sample with analytic and intuitive thinking during the CRT as additional covariate regressor

	Analytical Thinking					Intuitive Thinking				
	β	B	SE	t	P	β	B	SE	t	P
**Interventionist Belief**										
IL-Pat	0.43	3.22	0.81	3.98	<.001	0.43	3.26	0.80	4.06	<.001
Schizotypy	0.01	0.06	0.54	0.12	0.906	0.02	0.09	0.53	0.16	0.872
Parents' Belief	0.32	0.13	0.04	2.96	0.004	0.31	0.13	0.04	2.83	0.006
CRT Performance	-0.03	-0.02	0.09	-0.24	0.813	0.10	0.09	0.10	0.90	0.373
**Belief Change**										
IL-Pat	0.40	9.88	2.96	3.34	0.001	0.39	9.67	2.95	3.28	0.002
Schizotypy	0.02	0.36	1.96	0.18	0.855	0.02	0.28	1.96	0.14	0.886
Parents' Belief	-0.09	-0.12	0.16	-0.74	0.464	-0.08	-0.11	0.16	-0.67	0.503
CRT Performance	0.08	0.22	0.34	0.63	0.531	-0.10	-0.31	0.37	-0.84	0.407
**Universal Order**										
IL-Pat	0.38	11.64	3.61	3.23	0.002	0.37	11.44	3.61	3.16	0.002
Schizotypy	0.12	2.53	2.34	1.06	0.292	0.12	2.51	2.40	1.05	0.299
Parents' Belief	0.16	0.27	0.20	1.37	0.177	0.15	0.25	0.20	1.27	0.209
CRT Performance	0.09	0.32	0.42	0.78	0.439	-0.02	-0.08	0.45	-0.17	0.868

Supplementary References

1. Warren, Z. *et al. Afghanistan in 2014: A survey of the Afghan people*. (Asia Foundation, 2014).
2. Peer, E., Brandimarte, L., Samat, S. & Acquisti, A. Beyond the Turk: Alternative platforms for crowdsourcing behavioral research. *J. Exp. Soc. Psychol.* **70**, 153–163 (2017).
3. Palan, S. & Schitter, C. Prolific.ac—A subject pool for online experiments. *J. Behav. Exp. Finance* **17**, 22–27 (2018).
4. Levenson, H. Reliability and Validity of the I, P, and C Scales-A Multidimensional View of Locus of Control. (1973).
5. Bressan, P. The connection between random sequences, everyday coincidences, and belief in the paranormal. *Appl. Cogn. Psychol.* **16**, 17–34 (2002).
6. Rawlings, D. An exploratory factor analysis of Hartmann's Boundary Questionnaire and an empirically-derived short version. *Imagin. Cogn. Personal.* **21**, 131–144 (2001).
7. Eckblad, M. & Chapman, L. J. Magical ideation as an indicator of schizotypy. *J. Consult. Clin. Psychol.* **51**, 215 (1983).
8. Kelemen, D., Rottman, J. & Seston, R. Professional physical scientists display tenacious teleological tendencies: Purpose-based reasoning as a cognitive default. *J. Exp. Psychol. Gen.* **142**, 1074 (2013).
9. Plante, T. G. & Boccaccini, M. Reliability and validity of the Santa Clara strength of religious faith questionnaire. *Pastor. Psychol.* **45**, 429–437 (1997).
10. Cohen, A. B., Shariff, A. F. & Hill, P. C. The accessibility of religious beliefs. *J. Res. Personal.* **42**, 1408–1417 (2008).

11. Straube, B. & Chatterjee, A. Space and time in perceptual causality. *Front. Hum. Neurosci.* **4**, 28 (2010).
12. White, P. A. Visual impressions of pushing and pulling: The object perceived as causal is not always the one that moves first. *Perception* **41**, 1193–1217 (2012).
13. White, P. A. & Milne, A. Phenomenal causality: Impressions of pulling in the visual perception of objects in motion. *Am. J. Psychol.* **110**, 573 (1997).
14. Vandenberg, S. G. & Kuse, A. R. Mental Rotations, a Group Test of Three-Dimensional Spatial Visualization. *Percept. Mot. Skills* **47**, 599–604 (1978).
15. Barnes, K. & Gibson, N. J. S. Supernatural Agency: Individual Difference Predictors and Situational Correlates. *Int. J. Psychol. Relig.* **23**, 42–62 (2013).
16. Reed, P. *et al.* Seeing non-existent events: Effects of environmental conditions, schizotypal symptoms, and sub-clinical characteristics. *J. Behav. Ther. Exp. Psychiatry* **39**, 276–291 (2008).
17. Brugger, P. *et al.* 'Meaningful' Patterns in Visual Noise: Effects of Lateral Stimulation and the Observer's Belief in ESP. *Psychopathology* **26**, 261–265 (1993).
18. Ingersoll-Dayton, B., Krause, N. & Morgan, D. Religious trajectories and transitions over the life course. *Int. J. Aging Hum. Dev.* **55**, 51–70 (2002).
19. Meddin, J. R. Dimensions of Spiritual Meaning and Well-Being in the Lives of Ten Older Australians. *Int. J. Aging Hum. Dev.* **47**, 163–175 (1998).
20. Melia, S. P. Continuity in the Lives of Elder Catholic Women Religious. *Int. J. Aging Hum. Dev.* **48**, 175–189 (1999).
21. Shenhav, A., Rand, D. G. & Greene, J. D. Divine Intuition: Cognitive Style Influences Belief in God: (519702015-023). (2011) doi:10.1037/e519702015-023.

22. Jacowitz, K. E. & Kahneman, D. Measures of anchoring in estimation tasks. *Pers. Soc. Psychol. Bull.* **21**, 1161–1166 (1995).

23. Myers, S. M. An interactive model of religiosity inheritance: The importance of family context. *Am. Sociol. Rev.* 858–866 (1996).
