# Supplementary material for: Implicit pattern learning predicts individual differences in belief in God in the United States and Afghanistan
Source: Nat Commun. 2020 Sep 9;11:4503. doi: 10.1038/s41467-020-18362-3 (PMC7481241; doi:10.1038/s41467-020-18362-3)
Supplement: Supplementary file 3 — Reporting Summary [file 41467_2020_18362_MOESM3_ESM.pdf]

nature research

Corresponding author(s): Adam Weinberger and Adam Green

Last updated by author(s): Aug 2, 2020

Reporting Summary

Nature Research wishes to improve the reproducibility of the work that we publish. This form provides structure for consistency and transparency in reporting. For further information on Nature Research policies, see Authors & Referees and the Editorial Policy Checklist.

Statistics

For all statistical analyses, confirm that the following items are present in the figure legend, table legend, main text, or Methods section.

n/a	Confirmed	
☐	☒	The exact sample size (n) for each experimental group/condition, given as a discrete number and unit of measurement
☐	☒	A statement on whether measurements were taken from distinct samples or whether the same sample was measured repeatedly
☐	☒	The statistical test(s) used AND whether they are one- or two-sided<br>*Only common tests should be described solely by name; describe more complex techniques in the Methods section.*
☐	☒	A description of all covariates tested
☐	☒	A description of any assumptions or corrections, such as tests of normality and adjustment for multiple comparisons
☐	☒	A full description of the statistical parameters including central tendency (e.g. means) or other basic estimates (e.g. regression coefficient) AND variation (e.g. standard deviation) or associated estimates of uncertainty (e.g. confidence intervals)
☐	☒	For null hypothesis testing, the test statistic (e.g. F, t, r) with confidence intervals, effect sizes, degrees of freedom and P value noted<br>*Give P values as exact values whenever suitable.*
☒	☐	For Bayesian analysis, information on the choice of priors and Markov chain Monte Carlo settings
☒	☐	For hierarchical and complex designs, identification of the appropriate level for tests and full reporting of outcomes
☐	☒	Estimates of effect sizes (e.g. Cohen's d, Pearson's r), indicating how they were calculated

Our web collection on statistics for biologists contains articles on many of the points above.

Software and code

Policy information about availability of computer code

Data collection	Data was collected using Qualtrics and E-Prime.
Data analysis	All analyses were performed in STATA 15. Visualizations for Fig 2. performed with R.

For manuscripts utilizing custom algorithms or software that are central to the research but not yet described in published literature, software must be made available to editors/reviewers. We strongly encourage code deposition in a community repository (e.g. GitHub). See the Nature Research guidelines for submitting code & software for further information.

Data

Policy information about availability of data

All manuscripts must include a data availability statement. This statement should provide the following information, where applicable:

- Accession codes, unique identifiers, or web links for publicly available datasets
- A list of figures that have associated raw data
- A description of any restrictions on data availability

The study data are available on the OSF repository (https://osf.io/g5ywe/)

Field-specific reporting

Please select the one below that is the best fit for your research. If you are not sure, read the appropriate sections before making your selection.

☐ Life sciences ☒ Behavioural & social sciences ☐ Ecological, evolutionary & environmental sciences

For a reference copy of the document with all sections, see nature.com/documents/nr-reporting-summary-flat.pdf

Behavioural & social sciences study design

All studies must disclose on these points even when the disclosure is negative.

Study description	In this experiment, we tested the hypotheses that individual differences in implicit pattern learning are associated with differences in belief in an intervening/ordering God, changes in strength of belief over the lifespan, and intuitions of universal order.
Research sample	The U.S. sample was made up of 199 participants recruited from Georgetown University and the local community (Mage = 19.83 ±2.72 years, 65.83% female, 34.17% male). The Afghanistan sample was made up of 144 participants recruited from Karte Seh, Karte Sengi, and Dast-e Barchi sections of Kabul (Mage = 26.99 ±4.57 years, 41.22% female, 58.78% male). The European sample was made up of 96 participants recruited online from Prolific (Mage = 28.21 ±9.31 years, 83.33% European, 61.46% female, 37.50% male). We sought to collect samples from these populations due to the substantial religious and socio-cultural distinctness from (and sometimes opposition to) each other. Collecting samples that were distinct in these ways provided for a strong test of our hypotheses related to effects of implicit learning on religious belief, allowing us to evaluate the extent to whether any such effects might be general or context specific. We believe these samples are representative of the relevant populations, and distinct from one another as intended. Collecting independent samples also serves the more general purpose of enabling us to test the replicability of our results.
Sampling strategy	U.S. participants were recruited for $20 or course-credit (convenience sampling). Afghan participants were recruited through social networks by referral, following recommended practices for the study of culturally sensitive topics in Afghanistan (Warren et al., 2014). We sought to obtain relatively large samples in the U.S. and especially in Afghanistan because we anticipated that there might be substantial data loss (based on prior experience with data collection in Afghanistan; Warren et al., 2014). It was not possible to closely estimate likely effect sizes for our primary research questions a priori – we are not aware of prior empirical work investigating associations between implicit learning (or any unconscious process) and religious belief. Likewise, the precise amount of participant loss was not readily predictable. Rough power calculations (G*Power; Faul et al., 2007) indicated that a sample in the general range of 150 would be desirable, so our target recruitment numbers were determined in order to make final samples in that range likely, even after accounting for substantial participant loss.
Data collection	All belief and personality assessments, as well as demographic information, were collected through surveys on Qualtrics, which automatically logged responses. The Serial Reaction Time Task (SRTT) was presented in E-Prime, which also logged all participant responses. Participants completed all measures in a private room. All task elements were administered by Georgetown University (in the US Sample) and Afghan Cultural Center (in the Afghan sample) research assistants trained in the study protocol.<br><br>There were no experimental conditions in this study, and thus no blinding to condition, but researchers/task administrators did not have access to participants' responses during the study sessions.
Timing	All data were collected from March, 2015 to January, 2016, without any notable gaps. Following reviewer/editor feedback, we collected an additional online sample in April 2020 ("European sample"). We also re-contacted all U.S. participants ("U.S. re-contact sample) in January-March 2020.
Data exclusions	Prior to data collection, we anticipated that we may need to remove a number of participants – particularly from the Afghanistan sample – because of issues pertaining to cross-cultural relevance and sensitivity about religious material. The exclusion criteria for the Afghanistan sample were consistent with those established before data collection (with guidance provided by Z.W.). All participants were also required to be 18 years of age or over. In the U.S. sample, we followed previous lab guidelines. Experimenter notes were reviewed for all participants. Quality control of online data also followed previous approaches used by our lab (e.g., Weinberger et al., 2016). Participants were excluded if experimenter notes indicated a serious issue during the data collection.<br><br>Extensive QC/exclusion information is described in Supplementary Information 1:<br><br>Substantial data loss/exclusion for the Afghan sample was anticipated because issues related to cross-cultural relevance and comprehension are common for data collection in Afghanistan (Warren et al., 2014). Local experimenters were hired to conduct data collection sessions with local participants in Kabul, consistent with best practices based on Z.W.'s extensive experience collecting data in Afghanistan, e.g., as director of the Asia Foundation's Survey of the Afghan People1. Also consistent with best practices, we implemented additional post-collection quality control protocols to ensure that all data were properly collected and recorded by the local experimenters. This involved screening of the data to identify any irregularities associated with individual experimenters, reviewing experimenter notes for problems with study participants, and interviews with experimenters after the study to determine whether participants properly understood tasks, and whether the experimenters themselves had properly understood and implemented all study procedures.<br>We received data for 354 participants in Afghanistan. The majority of data collection occurred on site at the Afghan Cultural House in Kabul. Upon reviewing the data, we identified irregularities in data collection for two local experimenters (survey and task responses collected by these experimenters showed very low variability within and across participants, and response times were consistently different from the rest of the sample). Because these two experimenters were not able to satisfactorily account for their data, we excluded all 157 participants from whom these two experimenters collected data (all during the final months of the collection period). Two additional participants were excluded because notes at the time of the study indicated that they were responding dishonestly and/or that they were uncooperative with study procedures (e.g., one participant expressed unwillingness to cooperate because they perceived that they were "working with Westerners").<br>Among the 195 remaining participants, 8 were excluded from analyses for failing to finish the survey (i.e., they did not reach the end of the survey) and 9 were removed for improper completion times on the survey measures (defined as < 25 minutes or > 4 hours). An additional 14 participants were excluded because of inappropriate responding during the SRTT (defined as average RTs on the SRTT of >2000ms or <100 ms). In accordance with principles of informed consent and the Georgetown University institutional review board, participants were permitted to skip questions as desired. Sixteen participants did not complete key measures related to the hypotheses addressed by the current study (i.e., SRTT, IB measures, covariate personality/belief variables), and were thus excluded from analyses. After these exclusions, the final Afghan sample was 148 participants.

In the U.S. sample, the initial cohort of 240 was reduced to 199 for the following reasons. Five participants were removed because they did not comply with protocol for the testing session. Five participants did not complete the SRTT and two participants did not complete all of the belief measures. An additional 29 participants experienced a malfunction in the stimulus presentation during the SRTT. Specifically, the first random block consisted of only 22 targets (rather than 50). Theoretically, the reduced number of trials and task duration might have diminished participants' opportunity to increase their speed of responding over the course of the block. These 29 participants were conservatively removed from the primary analyses reported in the main text.
The online sample (i.e., European sample, Supplementary Table 2) was recruited through Prolific. Recent evidence indicates that Prolific offers higher quality data (e.g., based on participant attention and honest responding) and a more diverse participant pool compared to alternative online research platforms, such as Amazon Mechanical Turk2,3. The survey contained two attention check questions embedded within the study measures (e.g., "Blue is virtually always the same color as orange?"). Twelve participants did not correctly answer both of these attention check questions and were excluded from analysis. One additional participant was excluded for being under 18 years of age at the time of the study. The constituency of this sample was primarily (83%) European (Supplementary Table 2).
In sum, the vast majority of all exclusions were related to either experimenter unreliability (in the Afghan sample) or a malfunction in stimulus presentation (in the U.S. sample). The impact of all of these decisions on the results reported in the main text are displayed in Supplementary Tables 10, 11. Results were largely unchanged across different inclusion/exclusion criteria, indicating that these inclusion/exclusion determinations did not substantially impact the observed findings.

Non-participation

No participants dropped out of the study. It was only a single visit.

Randomization

There were no group assignments for this study, thus there was no randomization. The data presented in this paper was collected as part of a larger battery of tasks, which in total took approximately 1.5 hours. Additional measures are described in Supplementary Information 2. Behavioral/perceptual tasks were counterbalanced, but always came after personality assessments (i.e., schizotypal thinking) and before religious and demographic surveys. Thus, as the present study reports results related to just one such perceptual task (the SRTT), the described task elements were presented in the same order for all participants.

Reporting for specific materials, systems and methods

We require information from authors about some types of materials, experimental systems and methods used in many studies. Here, indicate whether each material, system or method listed is relevant to your study. If you are not sure if a list item applies to your research, read the appropriate section before selecting a response.

Materials & experimental systems

n/a	Involved in the study
☒	☐ Antibodies
☒	☐ Eukaryotic cell lines
☒	☐ Palaeontology
☒	☐ Animals and other organisms
☐	☒ Human research participants
☒	☐ Clinical data

Methods

n/a	Involved in the study
☒	☐ ChIP-seq
☒	☐ Flow cytometry
☒	☐ MRI-based neuroimaging

Human research participants

Policy information about studies involving human research participants

Population characteristics

Age and gender were obtained. As described in the main text, we also collected measures of personality traits (unusual thinking), belief in universal order, and religious belief. Schizotypal thinking and parental religious belief were included as covariates.

Recruitment

U.S. participants (N = 199, Mage = 19.83 ±2.72 years, 65.83% female, 34.17% male, 52.26% Christian, 25.13% unaffiliated, all others < 4%) were recruited via flyers and postings on Georgetown's Research Volunteer website as well as Research-Match, an online resource to advertise the study to the surrounding community. Afghan participants (N = 148, Mage = 26.99 ±4.57 years, 41.22% female, 58.78% male, religious affiliation not queried due to potential risks associated with non-Islamic affiliation) were recruited through social networks by referral, following recommended practices for the study of culturally sensitive topics in Afghanistan. While there is the potential for self-selection bias, the study was advertised in a very broad fashion ("Mind brain research", "Causal Perception and Cognitive Function Study"). This language did not explicitly mention religion, thus allowing for the enrollment of individuals with a range of religious beliefs. Georgetown and the surrounding area is predominantly Christian, and this is reflected in the religious information of our sample. Professing a non-Islamic faith in Afghanistan is dangerous. We did not ask for religious information in this sample, but participants were presumed to be Muslim. This difference between samples was an intended part of the study design as it allows for more universal claims regarding the relationship between visual-perceptual biases and religious belief. European sample participants were recruited online through Prolific.

Ethics oversight

All procedures in the U.S. and Afghanistan were approved by the Georgetown University IRB.

Note that full information on the approval of the study protocol must also be provided in the manuscript.
